# Accumulation of Phenolic Acids during Storage over Differently Handled Fresh Carrots

**DOI:** 10.3390/foods9101515

**Published:** 2020-10-21

**Authors:** Jarkko Hellström, Daniel Granato, Pirjo H. Mattila

**Affiliations:** 1 Production Technologies, Natural Resources Institute Finland (Luke), Myllytie 1, FI-31600 Jokioinen, Finland; jarkko.hellstrom@luke.fi; 2 Production Technologies, Natural Resources Institute Finland (Luke), Tietotie 2, FI-02150 Espoo, Finland; daniel.granato@luke.fi; 3 Production Technologies, Natural Resources Institute Finland (Luke), Itäinen Rantakatu 4, FI-20520 Turku, Finland

**Keywords:** phenolic acids, food processing, minimally processed foods, UHLPC-MS/MS

## Abstract

Carrots contain a significant content of phenolic compounds, mainly phenolic acids. Technological processing of carrots inflicts wounding stress and induces accumulation of these compounds, especially caffeic acid derivatives, in the periderm tissue. In this study, the effect of minimal processing (polishing, washing, peeling, and grating) on the retention of soluble phenolic acids in carrots was monitored during cold storage. Storage for up to 4 weeks and 24 h was used for whole and grated carrot samples, respectively. Total phenolic acid levels found in differently processed carrots varied greatly at the beginning of the storage period and on dry weight basis they ranged from 228 ± 67.9 mg/kg (grated carrot) to 996 ± 177 mg/kg (machine washed). In each case, processing followed by storage induced phenolic acid accumulation in the carrots. At the end of the experiment (4 weeks at +8 °C), untreated and machine-washed carrots contained ca. 4-fold more phenolic acids than at day 0. Similarly, polished carrots contained 9-fold and peeled carrots 31-fold more phenolic acids than at day 0. The phenolic acid content in grated carrot doubled after 24 h storage at +4 °C. Individual phenolic acids were characterized by high resolution mass spectrometry. MS data strongly suggest the presence of daucic acid conjugates of phenolic acids in carrot. Storage time did not have statistically similar effect on all compounds and generally in a way that dicaffeoyldaucic acid had the highest increase. This research provides important information for primary production, packaging, catering, the fresh-cut industry and consumers regarding the selection of healthier minimally processed carrots.

## 1. Introduction

It is well known that fruits, berries and vegetables are important components of a healthy diet. Vegetables contain several phenolic compounds which are recognized as antioxidants and possess many beneficial health effects, for example, in reduction of the risk of cardiovascular diseases, cancers, neurodegenerative diseases, diabetes and osteoporosis [1,2,3]. In foods, phenolic compounds may contribute to bitterness, astringency, color, flavor and oxidative stability of products [4]. Carrots are among the richest vegetable sources of phenolic acids. The main phenolic acid aglycone in carrots is caffeic acid, of which the share is over 70% of all phenolic acid aglycones. Caffeic acid occurs in carrot in the soluble forms of caffeoylquinic acids. Other phenolic acid aglycones in carrots are *p*-OH-benzoic, ferulic, vanillic and *p*-coumaric acids [5].

Due to changes in consumer behavior, the demand for minimally processed fruit and vegetable products has increased dramatically in recent years. Fresh carrots are currently available as untreated, washed, polished, peeled, grated and fresh cut. Due to the widespread diversity of the fresh carrot products there is a need to understand the retention of phytochemicals during processing and storage [6].

Several research groups have stated that the content of phenolics and the antioxidative capacity of carrots subjected to minimal processing could increase during storage [7,8,9,10,11,12,13,14,15,16]. Most of these studies have followed the concentration of only a few or one phenolic acid or even the total phenolic content [17]. Becerra-Moreno et al. [9] studied the effect of glyphosate on the accumulation on the individual phenolic acids in wounded (shredded) carrots, while Klaiber et al. [13] studied influence of washing treatment on individual phenolic acid contents of carrot sticks. Simões et al. [16] studied the effect of storage of carrots under O_2_ and CO_2_ levels on the individual phenolic acid contents and Viacava et al. [18] studied the effect of wounding stress and extrusion on the free and bound phenolic profiles. However, it seems that there is still no consensus on the profile of phenolic acids in carrot, and more data on the effect of various commercial and catering processes on the content of individual phenolic acids are also needed.

In fact, the accumulation of phenolic compounds in carrot has been found to be linked with the wound-induced activation of phenylalanine ammonia-lyase (PAL) synthesis with the purpose to repair the wounding damage and to prevent invasion by pathogens [12]. These processes could occur between a few minutes to several hours after wounding [19]. This mechanism is dependent on several factors, including wounding intensity, initial levels, cut methods and temperature, for example [1,14,20]. Currently, polishing is one of the most used methods to process fresh carrots for the retail markets. The polishing system includes washing and mild brushing, and it is designed to remove the surface membrane (periderm) from carrots. The shelf life of the polished carrots is much longer than that of washed or peeled. Traditional methods of washing leave this membrane layer intact and as it dries out, it turns opaque leaving the appearance of carrots unpleasant.

Although several studies have been published about the effects of wounding over phenolic accumulation in carrot, to our knowledge, information about the effect of polishing and peeling is not available or scarce, respectively. In addition, inadequate information is available about the effect of wounding and storage on the intact phenolic acid compounds. The compartment of various commercial and catering processes such as polished, peeled, untreated, machine washed, peeled and shredded carrots would be valuable. Hence, our goal was to study the effect of various treatments on the accumulation of the intact soluble phenolic acids during storage of carrots. As the consumption of fresh minimal processed vegetable products increases, the potential to enhance the phenolic acid content and intake of these health-promoting compounds is tempting. This research provides important information for primary production, packaging, catering, fresh-cut industry and consumers towards the selection of healthier minimally processed carrot.

## 2. Materials and Methods

### 2.1. Samples

Harvest-fresh carrot samples (*Daucus carota*, cv. Panther) were provided from the local commercial farmer, packer and marketer, Karotia Ltd. Three kinds of samples were collected, namely untreated (harvest-fresh), machine washed and machine washed and polished carrots. All carrot samples were from the same batch, and they were approximately the same size. A portion of the washed carrots was further processed in a laboratory by peeling using a household peeling knife. The peels were also collected for phenolic acid analysis. A portion of the peeled carrots was grated using a household food processor (Philips, Andover, MA, USA) in the laboratory.

**Figure 1:**
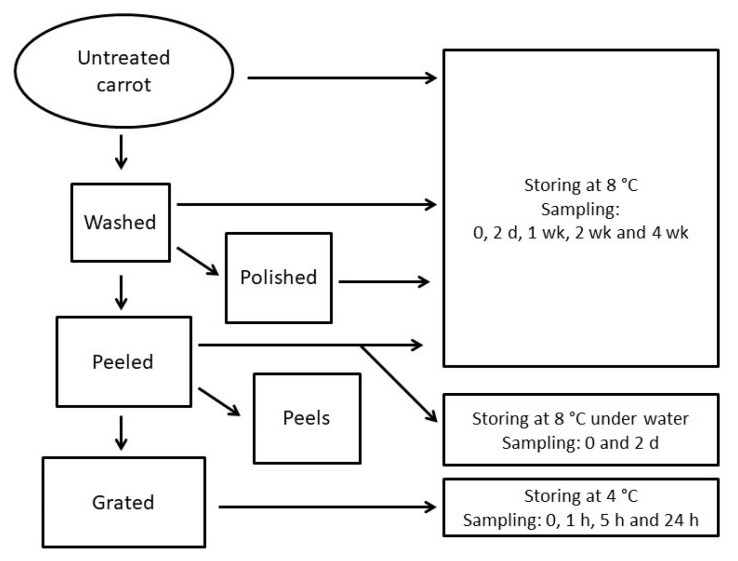
Scheme of carrot processing.

Untreated, washed, washed and polished and washed and peeled carrots were packed into retail plastic low-density polyethylene bags (LDPE; 0.5 kg, approximately 6–8 carrots/one plastic bag) and were stored at 8 °C in the dark cold storage. Samples for phenolic acid analysis were taken after 0, 2, 7, 14- and 28-days of storage (three replicate bags from all sample types, from all time points). Peeled carrots were stored also under water at 8 °C and samples were taken after 0 and 2 days (three 0.5 kg replicates from every time point). At different time points, the whole carrots from every sample replicate were halved lengthwise, and the halves were chopped, mixed and freeze-dried prior to the phenolic acid analysis. Grated carrot was stored at 4 °C in three bowls covered with aluminum foil, all containing 1.5 kg, and 100 g-samples were taken from all bowls after 0, 1, 5- and 24-h storage to be freeze-dried before analyses. Prior to freeze-drying the samples were frozen at −21 °C. Freeze-drying was carried out at −40 °C under vacuum of 0.1–0.2 mbar. A schematic representation of the study protocol is shown in Figure 1.

### 2.2. Quantification of Soluble Phenolic Acids

Freeze dried samples (0.5 g) were homogenized in 7 mL of a mixture of methanol, containing 2 g/L of butylated hydroxyanisole (BHA) obtained from Acros Organics (Geel, Belgium) and 10% acetic acid (85:15 *v*/*v*) with an IKA Ultra-Turrax T 25 homogenizer (IKA Werke GmbH & Co., Staufen, Germany). The homogenized sample extract was ultrasonicated for 30 min and increased to a volume of 10 mL with distilled water. After mixing, 1 mL of the solution was filtered (Acrodisc GHP, 0.2 μm, Port Washington, NY, USA). The analytical UHPLC system consisted of an Agilent 1290 Infinity Series ultra-high-performance liquid chromatograph (Agilent Technologies, Santa Clara, CA, USA) equipped with a diode array detector. Phenolic acid separation was done with a Zorbax Eclipse Plus C_18_ (2.1 × 50 mm, 1.8 μm) column (Agilent Technologies Inc., Santa Clara, CA, USA) with a C_18_ guard column. The temperature of the column oven was set at 35 °C. A gradient elution was employed with a mobile phase consisting of 50 mM H_3_PO_4_ at pH 2.5 (solution A) and acetonitrile (solution B) as follows: Isocratic elution 95% A, 0–1.2 min; linear gradient from 95% A to 85% A, 1.2–4.25 min; linear gradient from 85% A to 80% A, 4.25–10 min; linear gradient from 80% A to 50% A, 10–15 min; isocratic elution 50% A, 15–16.2 min; linear gradient from 50% A to 95% A, 16.2–17 min; post-time 2 min before the next injection. The flow rate of the mobile phase was 0.4 mL/min, and the injection volume was 2.0 μL. UV spectra of peaks were recorded between 190 and 400 nm. Chlorogenic acid (5-caffeoyl quinic acid) was used as an external standard for all caffeoyl quinic acids, caffeic acid for all other caffeic acid derivatives and ferulic acid for all ferulic acid derivatives with the assumption that the molar absorptivity at the detection wavelength (329 nm) depended solely on the structural cinnamic acid part in each compound. The results were then calculated according to the molecular masses of the actual compounds. All quantifications were based on peak area and the samples were analyzed in triplicate. In order to calculate the results in dry weight basis the residual moisture of freeze-dried carrots was determined by drying at 105 °C overnight (17 h).

### 2.3. Mass Spectrometric Identification

An Acquity UPLC—Xevo G2 QTOF high resolution mass spectrometer (Waters, Milford, MA, USA) operated by Waters MassLynx 4.1 software was used for the identification of phenolic acids using the analytical conditions as follows: Compounds were separated on Waters Acquity BEH C18 (1.7 µm, 2.1 mm × 150 mm) column using a gradient of 0.1% formic acid in H_2_O (A) and of 0.1% formic acid in acetonitrile (B). The gradient program was as follows: 2%–60% of B in 24 min, 60%–100% of B in 24–31 min, held at 100% of B for 2 min, 100%–2% in 1 min and held at 2% of B for 4 min. The flow rate was 0.55 mL/min, temperature of the column oven was 45 °C and the injection volume was 2 µl. An electrospray interface (ESI) was used with capillary voltage of −1 kV in negative mode. The sampling cone was set to 35 V and extraction cone to 4 V. The cone and desolvation nitrogen gas flows were 15 and 990 l/h, respectively. The desolvation temperature was 550 °C. Source temperature was 150 °C. Argon was used as the collision gas. MS analyses were done by data independent acquisition (MSE) centroid data mode in a full scan *m*/*z* 50–1200 with 0.2 sec scan time. In the MSE function, the precursor ions from the low-collision energy MS-mode were fragmented using high collision energy ramped up from 15 to 40 eV.

### 2.4. Statistical Analyses

Results were expressed as means followed by the standard deviation (*n* = 3). For inferential analysis, one-way ANOVA was used and, when applicable, Tukey’s test was applied to separate the means. The significance level was set to 0.05 to reject the null hypothesis (no difference between samples). TIBCO Statistica v. 13.3 (TIBCO Statistica, Palo Alto, CA, USA) was used in the analysis.

## 3. Results and Discussion

### 3.1. Identification and Quantification of Phenolic Acids

It is known that about 70% of carrot phenolics exist in soluble forms [5,18] and when carrots are treated with wounding stress, they produce high levels of caffeoylquinic acids, i.e., chlorogenic acid and its derivatives [13,14,20]. Hence, only soluble phenolic acids were studied in the present study. However, according to Viacava et al. [18] there is significant increase also in nonextractable phenolic acids during storage at 15 °C for 48 h.

**Figure 2:**
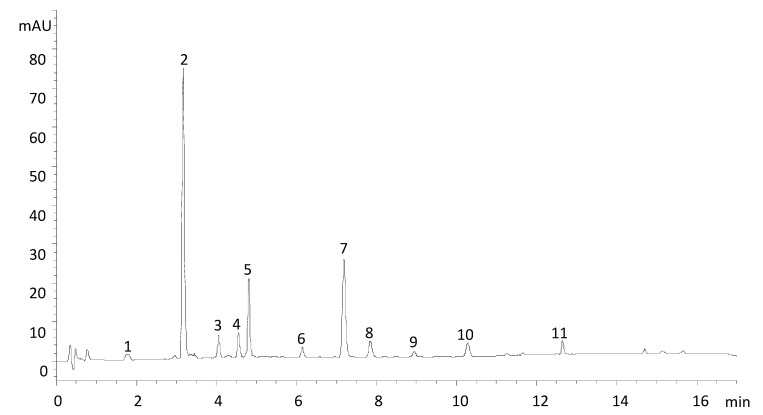
HPLC-DAD (λ = 329 nm) chromatogram of carrot extract. Peaks identified as 3-CQA (1), 5-CQA (2), FRut (3), FQA (4), CDA (5), 3,5-diCQA (6), diCDA (7), 4,5-diCQA (8), CFDA (9), FDA (10), diFDA (11).

Eleven different caffeic/ferulic acid derivatives were tentatively identified by UHPLC-DAD (Figure 2). They were further characterized by LC-QTOF-MS (Table 1) as 3-caffeoylquinic acid (3-CQA), 5-caffeoylquinic acid (5-CQA), feruloyl-rutinoside (FRut) caffeoyldaucic acid (CDA), feruloylquinic acid (FQA), dicaffeoyldaucic acid (diCDA), 3,5-dicaffeoylquinic acid(3,5-diCQA), 4,5-dicaffeoylquinic acid (4,5-diCQA), caffeoyl-feruloyldaucic acid (CFDA) and feruloyldaucic acid (FDA).

Accurate *m*/*z* values for the deprotonated caffeoylquinic acid isomers were 353.0867 and 353.0876 agreeing quite well with the theoretical monoisotopic mass (353.0873). The identification was further ensured by the MS^2^ fragments of 191.06 and 179.03 corresponding to quinic acid and caffeic acid, respectively. The later eluting CQA had the same retention time with the reference standard confirming it was 5-CQA while the elution order suggested that the earlier eluting CQA was 3-CQA [21]. Two dicaffeoylquinic acids were detected at *m*/*z* values of 515.1169 and 515.1182 (predicted *m*/*z* 515.1190) and MS^2^ fragments of 353.09, 191.05 and 179.03 corresponding to CQA, QA and CA, respectively. According to the retention order and previous studies [9,22] they were tentatively identified as 3,5-diCQA and 4,5-diCQA. Faisal et al. (2017) [23] characterized soluble phenolic acids in six carrot cultivars and all of them had 3-CQA, 5-CQA and two isomers of dicaffeoylquinic acid agreeing with our results. They also found two different feruloylquinic acid isomers in all cultivars while we could detect only one isomer (*m*/*z* 367.1042). Previous studies have also found 4-caffeoylquinic acid [24,25], diferuloylquinic acid [23] and caffeoylhexoside [26] in some carrot cultivars but we could not find them in our samples. However, a compound at *m*/*z* 501.1605 with a fragment at *m*/*z* 193.0491 (ferulic acid) was detected. These ions could be derived from feruloyl-rutinoside.

In the present study several daucic acid derivatives of hydroxycinnamic acids were tentatively identified in carrots, namely caffeoyldaucic acid, dicaffeoyldaucic acid, feruloyldaucic acid, diferuloyldaucic acid and caffeoyl-feruloyldaucic acid (Table 1). For diferolyldaucic acid the identification based solely on molecular ion since the signal was too weak for MS^2^.

**Figure 3:**
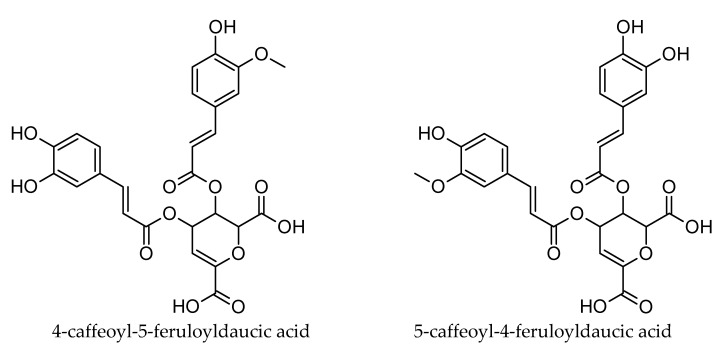
Two possible isomers of caffeoyl-feruloyldaucic acid.

Kammerer et al. [26], and a few years later Kreutzmann et al. [24], reported caffeic acid derivatives at *m*/*z* values of 365 and 527 in carrots. However, they did not provide any suggestions for the molecular or structural formulas. Recently, Pace et al. [27] tentatively identified those compounds as N-tryptophan conjugates. This conclusion is not supported by the HRMS data of the current study. For instance, a molecular ion at *m*/*z* 527.0816 has a much better match with a formula C_25_H_19_O_13_ (527.0826, deprotonated diDCA) than with C_26_H_27_N_2_O_10_ (527.1666, deprotonated caffeoyl-N-tryptophan-hexoside). Furthermore, the detected MS^2^ fragments 365.05, 203.02 and 179.04 are in good accordance with the structures of CDA, daucic acid, and caffeic acid, respectively. Dicaffeoyldaucic acid was for the first time isolated and fully characterized in sweet potato (Dini et al., 2006) [28] and later Toffali et al. [29] showed that, also, carrot cells can produce diCDA. In the same study, also, methylated diCDA was reported as a carrot metabolite. The MS^2^ fragment at *m*/*z* 193.05 detected in the present study indicates that the methylation appears most probably in caffeoyl moiety, i.e., the suggested compound at *m*/*z* 541.0969 is deprotonated caffeoyl-feruloyldaucic acid (Figure 3). Similarly, Kammerer et al. [26] reported a compound at *m*/*z* 541 with caffeoyl and feruloyl moieties. Albeit MS data strongly suggest that caffeic and ferulic acids in carrot appear to a considerable extent as daucic acid conjugates, further research, including NMR studies, is required to fully characterize these compounds.

### 3.2. Effect of Carrot Processing on Total Phenolic Acids

Total soluble phenolic acid levels (summed amount of phenolic acids with caffeoyl and feruloyl moieties) found in differently processed carrots varied much at the beginning of the storage on day 0 as calculated on a dry weight basis (Table 2). The dry weight of the whole and grated carrot samples varied from 11.4% to 12.5%, and the dry weight of carrot peels was 4.6%. At the beginning of the experiment untreated carrots contained total phenolic acids 893 ± 87 mg/kg DW (dry weight). Machine washing had no statistically significant effect (*p* > 0.05) on the contents (996 ± 177 mg/kg DW) and polishing of the carrots reduced the concentration moderately (666 ± 24 mg/kg DW, *p* < 0.05). Peeling had the most considerable effect, because total phenolic acid content of the peeled carrots was 246 ± 81 mg/kg DW at the beginning of the experiment, and this content did not appreciably diminish after grating (226 ± 68 mg/kg DW). The magnitude of these levels is in-line with earlier data [5,7]. The highest levels of phenolic acids were detected in peels (3270 ± 107 mg/kg DW). This was expected, because although phenolic acids are present in the root, they are mainly concentrated in the periderm [30]. According to Zhang and Hamauzu [31], although accounting for only 11% of the amount of the carrot fresh weight, peels could provide 54.1% of the amount of the phenolics in 100 g fresh weight of carrots.

**Figure 4:**
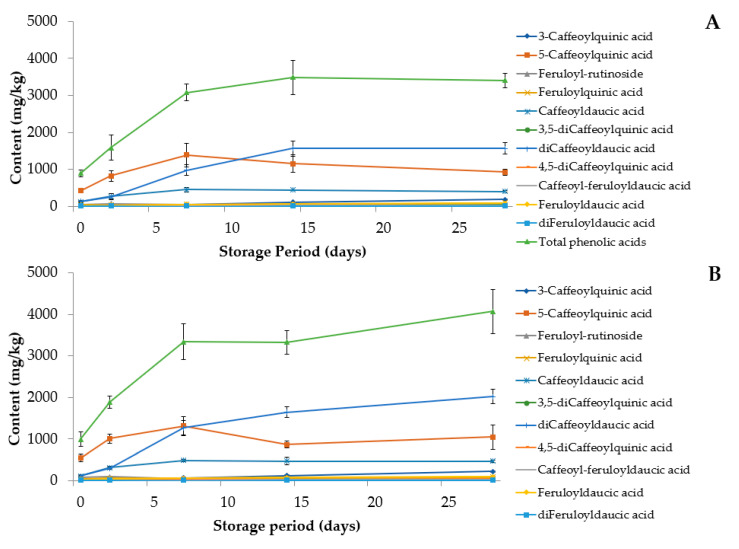

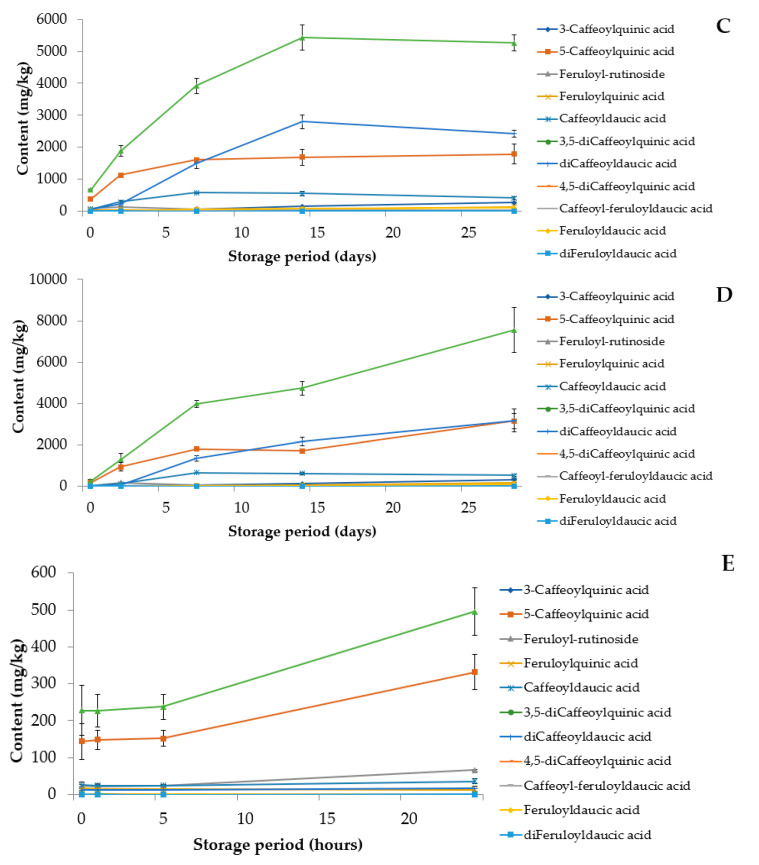
Phenolic acid composition (mg/kg DW) of untreated (**A**), washed (**B**), polished (**C**), peeled (**D**) and grated (**E**) carrots stored at 8 °C for either 28 days or 24 h.

Phenolic acids accumulated in all processed and the untreated carrots during cold storing at 8 °C. This was in accordance with several studies dealing with the induction of phenolic acids in wounded carrot products [7,8,9,10,11,12,13,14,15,16]. However, according to Alarcόn-Flores et al. [7] commercial fresh-cut carrots contained lower levels of phenolic acids than the fresh counterpart. Figure 4 shows that the induction of the total phenolic acids was very strong during the first week, after which more differences were found between the treatments. In the case of untreated carrots, the concentrations of total phenolic acids reached a plateau after two weeks of cold storing at 8 °C. The phenolic acid concentration of polished carrots increased linearly during the two days and two weeks cold storage and prolonging the storing from two to four weeks had statistically no further effect (*p* > 0.05) on the content of phenolic acids. In peeled carrots the phenolic acid contents differed statistically from each other at all time points (*p* < 0.05). The phenolic acid contents increased during the whole four-week storing period, reaching 7547 ± 1090 mg/kg DW, which implied a 31-fold increase. If the peeled carrots were stored under the water at the same temperature for 2 days, the content of phenolic acids (403 ± 72 mg/kg) was much lower than in carrots stored under normal atmosphere (1283 ± 306 mg/kg). This is probably since PAL activity is inhibited by low oxygen environments [30]. At the end of the experiment (four weeks), untreated and machine-washed carrots contained roughly 4-fold (3336 ± 191 and 3990 ± 506 mg/kg DW, respectively), and the polished carrots 8-fold (5256 ± 252 mg/100 g DW) more phenolic acids than at day 0. The phenolic acid content in grated carrot was not significantly changed during the first 5 h, but after 24 h storage the concentration was doubled (495 ± 64 mg/kg DW). This was in line with Viacava et al. [18] who found that total free and bound phenolic acid contents increased 288.1% and 407.6%, respectively, in shredded carrot, when stored at 15 °C for 48 h.

Although the peeled carrot seems to be a rich source of phenolic acids, the shelf life of fresh-cut carrots is strongly dependent on sensory quality which was estimated for these kinds of products to be only 4 days [6]. Instead, the use of gentle processes and optimal packaging technologies will lead to good quality products high in terpene flavor [32].

### 3.3. Effect of Carrot Processing on Individual Phenolic Acid Compounds

Chlorogenic acid was the main phenolic acid in all samples on day 0, which is in-line with previous reports [33,34]. Similarly, 5-CQA has been reported as the major caffeoylquinic acid in carrots [23,24,25,26,35,36], while some sources have indicated that 3-CQA is the most abundant form [22,27,37,38]. In the samples of the present study 3-CQA was always a minor phenolic acid. It is obvious that some variation should occur in the phenolic acid profile between different carrot varieties, but it is also very easy to confuse 3-CQA with 5-CQA due to the historical ambiguity in the nomenclature of such compounds [39]. IUPAC specified the rules for nomenclature of chemical compounds in 1976 and at that moment 3-CQA was turned to 5-CQA and vice versa. According to IUPAC, compounds known with their trivial names as chlorogenic acid and neochlorogenic acid are 5-CQA and 3-CQA, respectively. However, in the studies performed by Formica-Oliveira et al. [22] and Pace et al. [27], the terms chlorogenic acid and 3-CQA are used as synonyms and thus, it is plausible to assume that they were using the previous nomenclature and the compound in question was actually 5-CQA according to current rules. This would be very understandable since most of the chemical suppliers have kept the old, yet incorrect, nomenclature in their product catalogues [39]. After 5-CQA the next highest amounts were determined for daucic acid derivatives of caffeic acid (CDA and diCDA) in peels and untreated and washed carrots but not in polished, peeled or grated carrots (Table 2, Figure 4). Polishing and peeling removed the skin efficiently, so it seems that CDA and diCDA are especially concentrated in the skin of carrot.

The content of most phenolic acids increased in all treatments during storage for 28 days. The concentrations of diCDA increased more considerably—being 11-fold, 18-fold, 43-fold, and 392-fold in untreated, washed, polished and peeled carrot samples, respectively, after 28 days of storage (Figure 4). At the end of the storage period, diCDA was the main phenolic acid in all samples other than in peeled carrot, wherein the contents of diCDA and 5-CQA acid were of the same level. The concentrations of 5-CQA and CDA also increased significantly, especially in peeled carrot. In most treatments the most intense increase in 5-CQA and DCA content appeared during the first week after which they reached the plateau, or their content started to decrease slightly (Figure 4). For diCDA the period of intense accumulation was usually longer, i.e., two weeks and then plateau was reached. An exception was the peeled carrot where the content of 5-CQA and diCDA tended to increase the whole four weeks. A sharp increase was observed for 3-caffeoylquinic acid, from 10-fold (untreated carrot) to 138-fold (peeled carrot) although the concentrations remained lower compared with the main three compounds. It has been shown that wounding stress increases the contents of different CQAs during storage of carrots [40,41]. Surjadinata et al. [20] monitored the fate of phenolics during the storage of shredded carrot, and after one-week, the content of 5-CQA was 7.3–11.3-fold more than the original content. Accordingly, in the present study the content of 5-CQA in peeled carrot was ca. 11 times the initial content after one week storing.

Grated carrot is not expected to be stored for long periods due to its limited shelf life and hence, the accumulation of phenolic acids in grated carrot was followed only for 24 h in the present study. During the first 5 h, no changes in the phenolic composition were observed. After 24 h, the increase in the total phenolic acid content was almost entirely due to increase in the of 5-CQA. Accordingly, Becerra-Moreno et al. [41] and Viacava et al. [18] found that chlorogenic acid increased the most in shredded carrot. Interestingly, after 24 h next highest content after 5-CQA was found for the compound tentatively identified as feruloyl-rutinoside (FRut). In other carrot samples the FRut content seemed to be highest after two days storing after which it declined. It is possible that fast but temporary biosynthesis of FRut is among the first responses for wound-induced stress in carrot.

As soluble phenolic acids were converted to caffeic acid and ferulic acid equivalents it was evidential that caffeic acid was the most abundant phenolic acid aglycone in all samples (Table 3). This was in line with our previous study [5]. Additionally, the storage induced the biosynthesis of caffeic acid more remarkably than for ferulic acid. Increased biosynthesis of phenolic acids results from stress-induced increase in PAL activity as is well demonstrated in previous studies [12,42]. After four weeks of storage, untreated and machine-washed carrots contained roughly 5-fold, and the polished carrots 10-fold more caffeic acid compared to the content in the beginning of the storage (Table 3). In peeled carrots the caffeic acid content was over 40 times the initial content at the end of the experiment. The increase in ferulic acid content was much more modest being one and a half times the initial content in untreated and washed carrots, and two times and seven times the initial content in the polished and peeled carrots, respectively, at the end of the storing period. As a result, the proportion of caffeic acid increased from 82% or 85% to 94% or 97% of the combined caffeic and ferulic acid content during the experiment. In fact, an early study showed that the storage of carrots for 2 days increased the contents of caffeic acid derivatives in a more pronounced way compared to the ferulic acid content [41].

## 4. Conclusions

In conclusion, minimally processed carrots can be an inexpensive, rich source of phenolic acids in the diet. By simply increasing wounding stress intensity it is possible to enhance the biosynthesis of phenolic acids. The phenolic acid content of harvest-fresh carrots multiplies during a few days of storage. Peeling greatly reduces the immediate phenolic content of carrots but a few days of storage will increase it efficiently. However, polishing seems to be an especially good practice to process carrots for retail markets. The appearance and shelf life of polished carrots are good, and their phenolic acid content increases greatly during storage. During storage, the greatest increases were found in the concentrations of dicaffeoyldaucic acid and 5-calffeoylquinic acid. The exception was grated carrot in which the largest increases were found in the concentration of 5-calffeoylquinic acid and feroyl-rutinoside.

## Figures and Tables

**Table 1:** MS data of tentatively identified phenolic acids in carrot.

Phenolic Acid	Deprotonated Formula	Monoisotopic Mass	Detected Mass (*m*/*z*)	Mass Difference (ppm)	Characteristic Fragments (MS^2^)
3-Caffeoylquinic acid, 3-CQA	C_16_H_17_O_9_	353.0873	353.0876	0.85	191.0558, 179.0345
5-Caffeoylquinic acid, 5-CQA	C_16_H_17_O_9_	353.0873	353.0867	−1.70	191.0551, 179.0348
Feruloyl-rutinoside	C_22_H_29_O_13_	501.1608	501.1605	−0.60	193.0491
Caffeoyldaucic acid, CDA	C_16_H_13_O_10_	365.0509	365.0499	−1.37	203.0183, 179.0337
Feruloylquinic acid, FQA	C_17_H_19_O_9_	367.1029	367.1042	3.54	191.0556, 193,0514
Dicaffeoyldaucic acid, diCDA	C_25_H_19_O_13_	527.0826	527.0816	−1.90	365.0507, 203.0190, 179.0345
3,5-Dicaffeoylquinic acid, 3,5-diCQA	C_25_H_23_O_12_	515.1190	515.1169	−4.08	353.0897, 191.0536, 179.0354
4,5-Dicaffeoylquinic acid, 4,5-diCQA	C_25_H_23_O_12_	515.1190	515.1182	−1.55	353.0888, 191.0540, 179.0305
Caffeoyl-feruloyldaucic acid, CFDA	C_26_H_21_O_13_	541.0982	541.0969	−2.40	379.0662, 193.0504, 365.0496
Feruloyldaucic acid, FDA	C_17_H_15_O_10_	379.0655	379.0687	0.44	193.0848, 203.0158
Diferuloyldaucic acid	C_27_H_23_O_13_	555.1139	555.1155	2.88	ND

**Table 2:** Initial contents of phenolic acids in untreated, washed, polished, peeled and grated carrots (mg/kg DW).

Phenolic Acid	Carrot Sample					
	*Untreated*	*Washed*	*Polished*	*Peeled*	*Grated*	*Peels*
3-Caffeoylquinic acid	19.1 ± 2.57	14.7 ± 1.3	10.2 ± 1.8	2.35 ± 0.7	2.07 ± 0.6	57.0 ± 1.8
5-Caffeoylquinic acid	429 ± 45.5	540 ± 90.7	374 ± 20.0	164 ± 59.5	144 ± 48.2	1760 ± 55
Feruloyl-rutinoside	45.4 ± 5.7	83.1 ± 24.9	69.6 ± 6.8	20.4 ± 6.3	23.3 ± 4.0	261 ± 7.2
Feroyl-quinic acid	41.9 ± 9.7	52.0 ± 13.5	43.5 ± 6.5	24.5 ± 6.7	16.6 ± 5.3	163 ± 19.0
Caffeoyldaucic acid	121 ± 6.2	112 ± 21.5	64.7 ± 6.7	20.5 ± 7.3	25.2 ± 9.7	396 ± 13.3
3,5-diCaffeoylquinic acid	12.9 ± 0.8	11.7 ± 2.8	7.54 ± 0.7	1.75 ± 0.8	1.40 ± 0.1	42.2 ± 2.4
Dicaffeoyldaucic acid	137 ± 21.7	113 ± 24.0	56.2 ± 3.7	8.10 ± 1.9	11.9 ± 7.3	356 ± 12.7
4,5-diCaffeoylquinic acid	26.5 ± 2.0	21.6 ± 1.8	12.4 ± 1.9	1.04 ± 0.1	1.04 ± 0.5	76.7 ± 5.0
Caffeoylferuloyldaucic acid	8.30 ± 1.6	7.48 ± 1.4	5.16 ± 1.0	0.64 ± 0.1	0.64 ± 0.4	25.9 ± 0.4
Feruloyldaucic acid	34.6 ± 3.5	28.7 ± 3.6	15.9 ± 0.7	1.76 ± 0.3	1.21 ± 0.4	85.9 ± 4.2
diFeruloyldaucic acid	16.9 ± 2.1	12.2 ± 0.6	6.42 ± 0.4	0.96 ± 0.1	0.66 ± 0.0	46.4 ± 2.8
Total	893 ± 87.2	996 ± 177.2	666 ± 23.7	246 ± 81.4	227 ± 67.9	3270 ± 106.6

**Table 3:** Total caffeic and ferulic acid in carrot subjected to different technological procedures calculated as free acids.

Storage Period	Caffeic Acid (mg/kg)	Ferulic Acid (mg/kg)	Dry Matter (%)
Days	*Untreated Carrots*		
0	411 ± 42.8 ^b^	71.8 ± 4.9 ^b^	11.71 ± 0.49
2	777 ± 175.8 ^b^	70.5 ± 11.5 ^b^	12.15 ± 0.30
7	1657 ± 96.7 ^a^	79.3 ± 17.3 ^b^	11.81 ± 0.19
14	1970.2 ± 256.6 ^a^	80.64 ± 16.3 ^b^	11.68 ± 0.27
28	1898 ± 113.8 ^a^	117.6 ± 11.9 ^a^	11.46 ± 0.18
*p*-value ^1^	<0.001	0.008	
Days	*Washed Carrots*		
0	440 ± 74.4 ^b^	84.8 ± 17.3 ^a^	11.99 ± 0.28
2	916 ± 57.1 ^b^	90.2 ± 16.5 ^a^	12.22 ± 0.37
7	1839 ± 244.5 ^a^	77.3 ± 6.2 ^a^	11.57 ± 0.35
14	1901 ± 169.0 ^a^	86.6 ± 4.6 ^a^	12.10 ± 0.61
28	2318 ± 278.3 ^a^	117 ± 26.2 ^a^	11.63 ± 0.37
*p*-value ^1^	<0.001	0.100	
Days	*Polished Carrots*		
0	281 ± 5.3 ^d^	63.8 ± 5.9 ^b^	12.52 ± 0.23
2	891 ± 84.8 ^c^	87.7 ± 16.1 ^b^	11.99 ± 0.15
7	2170 ± 142 ^b^	73.4 ± 8.7 ^b^	11.85 ± 0.42
14	3150 ± 239 ^a^	94.2 ± 9.4 ^b^	11.97 ± 0.32
28	2963 ± 102 ^a^	145 ± 20.0 ^a^	11.89 ± 0.24
*p*-value ^1^	<0.001	<0.001	
Days	*Peeled Carrots*		
0	103 ± 35.7 ^c^	22.3 ± 6.0 ^c^	12.25 ± 0.19
2	562 ± 127.5 ^c^	75.7 ± 23.9 ^c^	11.90 ± 0.64
7	2200 ± 104.7 ^b^	45.3 ± 6.6 ^b^	11.78 ± 0.87
14	2722 ± 191.1 ^b^	53.4 ± 8.2 ^b^	11.73 ± 0.96
28	4256 ± 620.7 ^a^	146 ± 46.7 ^a^	11.93 ± 0.35
*p*-value ^1^	<0.001	<0.001	
Hours	*Grated Carrot*		
0	96.0 ± 35.0 ^b^	18.7 ± 2.0 ^b^	12.16 ± 0.32
1	97.5 ± 20.6 ^b^	16.6 ± 2.4 ^b^	12.28 ± 0.14
5	100 ± 14.5 ^b^	17.1 ± 2.2 ^b^	12.11 ± 0.26
24	200 ± 32.9 ^a^	33.9 ± 1.7 ^a^	12.08 ± 0.25
*p*-value ^1^	0.004	<0.001	

Note: ^1^ Probability values obtained by one-way ANOVA. Different letters in the same column represent different means (*p* < 0.05).
